# Early screening and post-treatment chronic endometritis in subsequent frozen embryo transfer cycles among women with first implantation failure: a retrospective cohort study

**DOI:** 10.3389/fendo.2026.1811073

**Published:** 2026-07-08

**Authors:** Longlong Wei, Yixuan Zhang, Shuna Wang, Siyue Xu, Weiran Hu

**Affiliations:** 1 Reproductive Medicine Department, People’s Hospital of Zhengzhou University, Zhengzhou, Henan, China; 2 Reproductive Medicine Department, Henan Provincial People’s Hospital, Zhengzhou, Henan, China

**Keywords:** Chronic endometritis, frozen-thawed embryo transfer, implantation failure, assisted reproductive technology, hysteroscopy, antibiotics

## Abstract

**Background:**

First implantation failure (FIF) remains a significant challenge in *in vitro* fertilization/intracytoplasmic sperm injection-embryo transfer (IVF/ICSI-ET), and a considerable proportion of affected women subsequently progress to recurrent implantation failure (RIF). Chronic endometritis (CE), an inflammatory disorder of the endometrium, is associated with implantation failure and poor reproductive outcomes. However, the clinical significance of varying CD138-positive plasma cell thresholds and the role of antibiotic therapy in women with FIF remain inadequately defined.

**Methods:**

A total of 4,528 women who experienced FIF following IVF/ICSI at Henan Provincial People’s Hospital were initially screened; of these, 2,885 women who underwent a subsequent frozen-thawed embryo transfer (FET) cycle were included in the final analysis. All participants underwent hysteroscopy-guided endometrial biopsy with CD138 immunohistochemical evaluation at the initial post-FIF assessment. Women diagnosed with CE received standardized empirical antibiotic treatment followed by post-treatment CD138 reassessment before the subsequent FET cycle. Multivariable logistic regression was performed to adjust for prespecified clinically relevant confounders. In addition, inverse probability of treatment weighting (IPTW) based on propensity scores was applied as a sensitivity analysis for the comparison between the PCE group and the CD138-positive/HPF ≤4 group. The primary outcome was live birth rate (LBR), and secondary outcomes included clinical pregnancy rate (CPR) and early miscarriage rate (EMR).

**Results:**

CE, defined as ≥5 CD138-positive plasma cells per high-power field (HPF), was identified in 16.8% of participants. The cure rate was 89.1% in women with CD138-positive plasma cells ≥5/HPF after two courses of standardized antibiotic treatment. No significant difference in pregnancy outcomes was observed between women with CD138-positive cell counts of 0/HPF and those with counts of 1-4/HPF. Women with CE who achieved histological remission (≤4/HPF on post-treatment biopsy) after treatment had pregnancy outcomes comparable to those of women with CD138-positive/HPF ≤4. Persistent chronic endometritis (PCE), defined as persistent ≥5 CD138-positive plasma cells per HPF after two standard antibiotic courses, was associated with lower LBR (28.3% vs. 41.8%, P = 0.034) and CPR (39.6% vs. 56.6%, P = 0.016) than in women with CD138-positive/HPF ≤4. In the IPTW-weighted analysis, baseline imbalance was substantially reduced, and PCE remained significantly associated with lower CPR (weighted OR 0.65, 95% CI 0.47-0.89, P = 0.008), while the association with lower LBR remained directionally consistent but attenuated to borderline significance (weighted OR 0.73, 95% CI 0.53-1.02, P = 0.056).

**Conclusion:**

Among women who have experienced FIF, an endometrial CD138-positive plasma cell count of ≤4/HPF is not associated with impaired pregnancy outcomes. Furthermore, following standard antibiotic therapy, women with ≥5 CD138-positive plasma cells/HPF had pregnancy outcomes comparable to those of women with ≤4 CD138-positive plasma cells/HPF. However, PCE was consistently associated with poorer reproductive outcomes, including in the IPTW-weighted analysis, although causal inference remains limited by the observational design.

## 1 Introduction

*In vitro* fertilization and intracytoplasmic sperm injection (IVF/ICSI) have become cornerstones of contemporary infertility management. Despite continuous advances in laboratory techniques and clinical protocols, the clinical pregnancy rate per transfer cycle remains around 40-50%, depending on factors such as maternal age, embryo quality, and transfer timing (1). Consequently, a substantial proportion of patients experience first implantation failure (FIF). Recurrent implantation failure (RIF) is typically diagnosed after multiple failed embryo transfers and should not be regarded as biologically equivalent to a single failed attempt. Importantly, not all women with an initial failed transfer will progress to true RIF. Because reproductive outcomes worsen as implantation failures accumulate, FIF may represent the earliest clinically recognizable stage at which potentially reversible etiologies can be identified before RIF becomes established (2–4).

However, existing research has primarily focused on women with established RIF, thereby overlooking potentially modifiable endometrial abnormalities at the earliest stage of implantation failure. In addition, recent data from RIF cohorts suggest that the prevalence of chronic endometritis (CE) identified using CD138-based diagnostic criteria is influenced by clinical confounders and diagnostic context, further supporting the need for stage-specific evaluation in women with FIF (5). This distinction is clinically important because FIF and RIF likely represent different stages along the implantation-failure spectrum, and earlier evaluation at the FIF stage may offer an opportunity to identify actionable endometrial factors before repeated failure becomes established (2–4).

Chronic endometritis (CE) is a persistent inflammatory condition of the endometrium, frequently associated with chronic microbial infection (6). Currently, immunohistochemical detection of CD138 (syndecan-1) is widely accepted as the standard and most reliable histopathological method for identifying CE (7). Nevertheless, a standardized diagnostic threshold for CD138-positive plasma cell counts has not yet been established. While some studies adopt a stringent threshold of ≥1 plasma cell per high-power field (HPF), others propose a cutoff of ≥5 cells/HPF (8, 9). This lack of consensus has contributed to considerable heterogeneity in reported prevalence estimates and variability in clinical management strategies.

CE has been consistently associated with impaired implantation outcomes (10, 11). From a mechanistic perspective, CE may disrupt the endometrial microenvironment through persistent inflammatory activation, immune dysregulation, stromal edema, abnormal uterine contractility, and impaired decidualization, thereby potentially compromising embryo implantation and subsequent placentation (12, 13).

Although antibiotic therapy has been reported to effectively eradicate CE and improve reproductive outcomes in women with RIF or recurrent pregnancy loss (RPL) (14, 15), the consistency and magnitude of this benefit remain variable across studies (11, 16). This inconsistency may be attributable to heterogeneous diagnostic criteria and limited sample sizes. Notably, although CE has been extensively investigated in women with RIF, the reported prevalence in that population varies substantially across studies, with representative cohorts reporting rates of 14.0% and 30.3% (8, 9). By contrast, CE-specific data in women with FIF remain sparse. Therefore, FIF should not be assumed to carry the same CE burden as established RIF; rather, its expected prevalence is more appropriately considered to lie within the lower-to-middle portion of the heterogeneous RIF spectrum and should be directly estimated in dedicated FIF cohorts.

Given that FIF represents the earliest clinically recognizable and potentially actionable stage in the implantation-failure trajectory, timely evaluation of CE at this phase provides an opportunity for earlier etiologic investigation and threshold-based management before repeated implantation failure becomes established. To our knowledge, this is one of the largest studies to date specifically evaluating the prevalence of CE and the potential role of antibiotic therapy in women experiencing FIF. This study aimed to inform a pragmatic, threshold-based management pathway for women with FIF by clarifying reproductive outcomes stratified by CD138-positive plasma cell counts and to examine the association between persistent chronic endometritis (PCE) and subsequent frozen-thawed embryo transfer (FET) outcomes.

## 2 Materials and methods

### 2.1 Study design and patient recruitment

**Figure 1:**
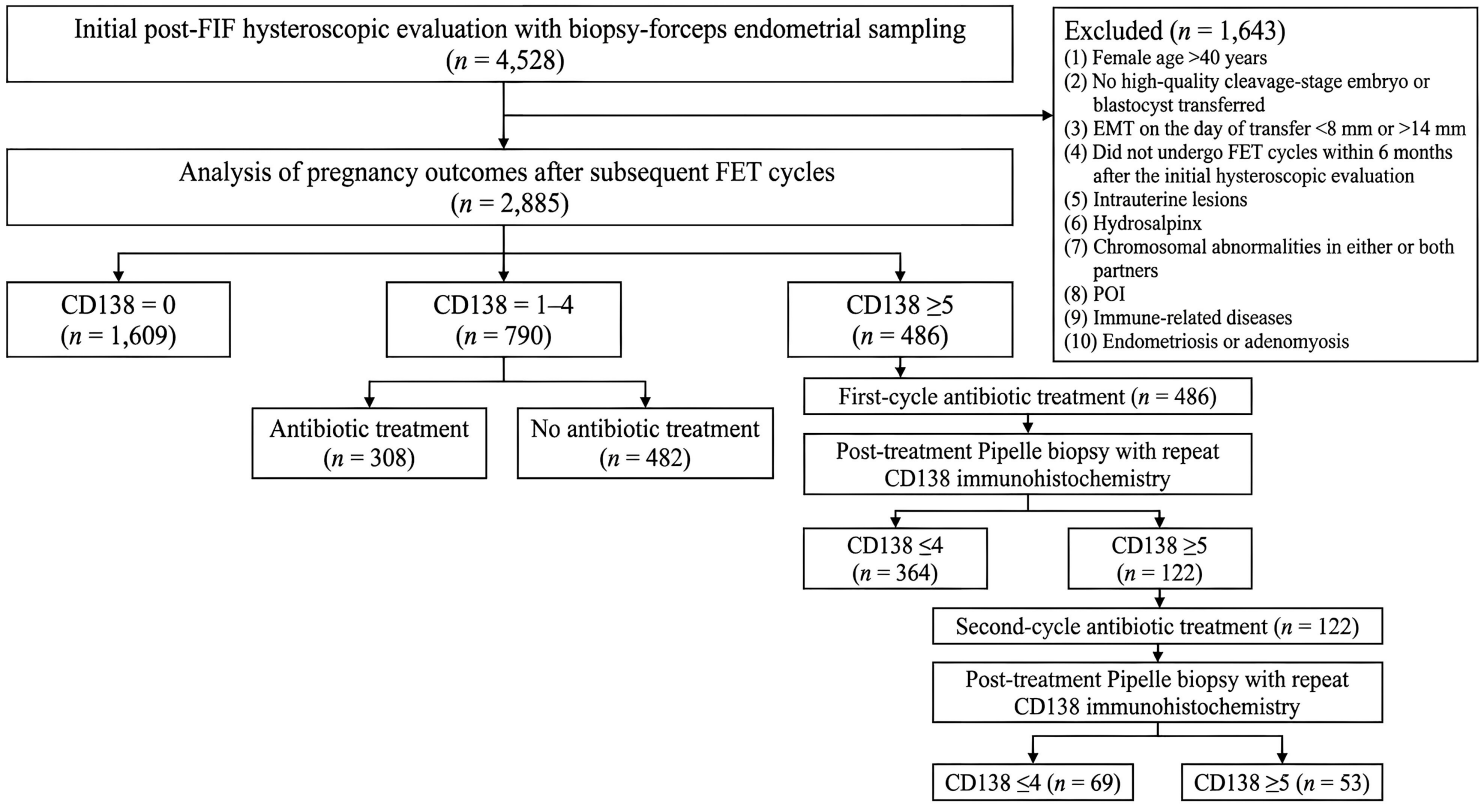
Study flowchart. CE, chronic endometritis; CCE, cured chronic endometritis after antibiotic treatment; PCE, persistent chronic endometritis after two courses of antibiotic treatment; FET, frozen-thawed embryo transfer; EMT, endometrial thickness; POI, primary ovarian insufficiency.

The study flowchart is shown in **Figure 1**. A total of 4,528 women who experienced first implantation failure following IVF/ICSI at Henan Provincial People’s Hospital between January 2021 and January 2025 were initially screened for eligibility. FIF was operationally defined as failure to achieve pregnancy after the first embryo transfer cycle, as indicated by a negative serum β-hCG result 14 days after embryo transfer, provided that at least one morphologically high-quality embryo was transferred in that index cycle. At our center, diagnostic hysteroscopy with endometrial biopsy was performed routinely as part of the post-FIF uterine cavity evaluation pathway, rather than being restricted to patients with a prior clinical suspicion of CE. Once CE was histologically confirmed according to the prespecified diagnostic criterion, antibiotic treatment was provided as part of routine clinical management. Withholding treatment from women with histologically confirmed CE solely to establish an untreated comparator group was deemed ethically impermissible in routine clinical practice. Subsequent reproductive outcomes were evaluated in the consecutive FET cycle. After application of the predefined exclusion criteria, 2,885 women who subsequently underwent FET were included in the final retrospective analysis. The exclusion criteria were as follows: (1) female age >40 years; (2) no high-quality embryo transferred in the index failed cycle; (3) endometrial thickness (EMT) on the day of transfer < 8 mm or > 14 mm; (4) no subsequent FET within six months after hysteroscopy; (5) intrauterine adhesions, uterine malformation, submucosal myoma, or intrauterine mass; (6) hydrosalpinx; (7) chromosomal abnormalities in either or both partners; (8) primary ovarian insufficiency; (9) immune-related diseases, such as systemic lupus erythematosus or ankylosing spondylitis; and (10) ultrasound-identified endometriosis or adenomyosis.

High-quality cleavage-stage embryos were defined according to the Istanbul consensus as day-3 embryos with 7-9 blastomeres, fragmentation ≤5%, and no multinucleation. High-quality blastocysts were defined as blastocysts graded 3BB, 4BB, or higher, excluding any blastocyst with a grade containing C (17). To avoid ambiguity, embryo-related variables used to define the index failed transfer were distinguished from those analyzed in the subsequent FET cycle. In the outcome analyses, the number of embryos transferred and the proportion of high-quality blastocysts transferred in the subsequent FET cycle were included as embryo-related clinical variables and adjustment factors.

The study protocol was approved by the Institutional Review Board of Henan Provincial People’s Hospital (Approval No. SYSZ-LL-2021091501). Informed consent was waived owing to the retrospective design, and all patient data were anonymized.

## 3 Diagnosis and treatment of CE

### 3.1 Diagnosis of CE

Hysteroscopy was performed during the follicular phase of the menstrual cycle as part of the initial post-FIF uterine cavity evaluation. Although hysteroscopy may provide visual findings suggestive of CE, the diagnosis of CE was based on histopathological examination rather than hysteroscopic appearance alone (18). At this initial diagnostic evaluation, endometrial tissue was obtained under direct hysteroscopic visualization using sterile biopsy forceps. The specimens were immediately immersed in 4% neutral-buffered paraformaldehyde for subsequent histopathological examination and CD138 immunohistochemical staining (7, 19). CE was defined as the presence of CD138-positive plasma cells ≥5/HPF in the endometrial stroma (21).

Post-treatment histological reassessment was performed separately from the initial diagnostic procedure. After antibiotic treatment, repeat endometrial sampling was performed using a Pipelle catheter during the proliferative phase before the subsequent FET cycle. Repeat CD138 immunohistochemical assessment of the Pipelle biopsy specimen was used to determine post-treatment CE status and to classify women with initial CE as having CCE or PCE.

### 3.2 Antibiotic treatment and follow-up of CE

Women diagnosed with CE, defined as CD138-positive plasma cells ≥5/HPF at the initial post-FIF evaluation, received standardized empirical antibiotic treatment as part of routine clinical management. Because persistent untreated CE may be associated with impaired reproductive outcomes, withholding treatment from women who met the prespecified histological criterion for CE solely for research comparison was considered ethically inappropriate. Accordingly, no untreated CE control group was available in this retrospective cohort.

The initial antibiotic regimen consisted of oral doxycycline at a dose of 100 mg twice daily for 14 days. After completion of the initial treatment course, repeat endometrial sampling using a Pipelle catheter was performed before the subsequent FET cycle for CD138 immunohistochemical reassessment. CCE was operationally defined as initial CE followed by post-treatment CD138-positive plasma cells <5/HPF, equivalent to ≤4/HPF, on repeat endometrial biopsy.

Women with persistent CD138-positive plasma cells ≥5/HPF after the initial antibiotic course received a second empirical regimen consisting of oral levofloxacin at a dose of 200 mg twice daily plus metronidazole at a dose of 500 mg three times daily for 14 days, followed by repeat Pipelle biopsy for histological reassessment. PCE was defined as persistent CD138-positive plasma cells ≥5/HPF after two consecutive antibiotic courses. This treatment strategy was protocol-based and was not guided by pathogen identification, antimicrobial susceptibility testing, or virological evaluation.

Given the ongoing controversy regarding the clinical significance of low-level plasma cell infiltration, management of women with CD138-positive plasma cells of 1-4/HPF was individualized according to clinician judgment and shared decision-making. Reproductive outcomes were evaluated in the subsequent FET cycle, which occurred within six months after the initial hysteroscopic evaluation according to the eligibility criteria.

### 3.3 Endometrial preparation regimens and frozen-thawed embryo transfer

Endometrial preparation followed three protocols: natural cycle (NC), hormone replacement therapy (HRT), or GnRH agonist-hormone replacement therapy (GnRH-a-HRT). In the NC protocol, follicular monitoring began on cycle days 10-12; FET occurred 3 days after ovulation for cleavage-stage embryos or 5 days after ovulation for blastocysts. In the HRT protocol, estradiol valerate (4-8 mg/day) was initiated on days 2-4; progesterone was initiated upon achievement of an endometrial thickness of ≥7 mm or when the thickness matched the peak thickness observed in previous cycles. FET was performed after 4 days of progesterone for cleavage-stage embryos or 6 days for blastocysts. In the GnRH-a-HRT protocol, triptorelin (3.75 mg) was administered on days 2-4; pituitary downregulation was confirmed by endometrial thickness <5 mm, LH <5 IU/L, estradiol <50 pg/mL, and absence of dominant follicles or ovarian cysts; estrogen supplementation was initiated 14-28 days later. Luteal support was standardized across all groups: vaginal progesterone gel (90 mg/day) plus oral progesterone (40 mg/day); estradiol therapy was continued in the HRT and GnRH-a-HRT groups.

### 3.4 Definitions of reproductive outcomes

Primary outcome: LBR, defined as the number of deliveries resulting in at least one live birth per 100 embryo transfer cycles. Secondary outcomes: CPR, defined as the number of pregnancies with ultrasonographic confirmation of a gestational sac per 100 embryo transfers; and EMR, defined as the proportion of spontaneous losses before 12 weeks’ gestation among clinical pregnancies.

### 3.5 Statistical analyses

Continuous variables were reported as median (interquartile range) and compared using the Mann-Whitney U test for two-group analyses or the Kruskal-Wallis test for comparisons across three groups. Categorical variables were analyzed using the chi-square test or Fisher’s exact test. Multivariable logistic regression analyses were performed to adjust for prespecified clinically relevant confounders, including maternal age, BMI, infertility duration, AMH level, endometrial preparation protocol, number of embryos transferred, EMT on transfer day, and the proportion of high-quality blastocysts transferred. Covariates were selected *a priori* based on clinical relevance. Results were reported as odds ratios (ORs) with 95% confidence intervals (CIs). Due to an insufficient number of early miscarriage events, multivariable modeling for EMR was not performed. As a sensitivity analysis for the comparison between the PCE group and the group with CD138-positive cell counts ≤4/HPF, propensity scores were estimated using a logistic regression model including maternal age, BMI, infertility duration, AMH, endometrial preparation protocol, number of embryos transferred, endometrial thickness on transfer day, and the proportion of high-quality blastocysts transferred. Stabilized inverse probability of treatment weighting (IPTW) was then applied to improve covariate balance between groups. Covariate balance before and after weighting was assessed using absolute standardized mean differences (SMDs), with an absolute SMD < 0.10 considered indicative of acceptable balance. Outcome analyses in the weighted cohort were performed using weighted logistic regression with robust variance estimation. Statistical significance was defined as a two-sided P value <0.05.

## 4 Results

### 4.1 Baseline characteristics of the study cohort

Among 4,528 women who underwent hysteroscopy-guided endometrial biopsy, 2,885 met the inclusion criteria and were retained in the final analytic cohort (**Figure 1**). Baseline characteristics are presented in **Tables 1**–**3**. In unadjusted comparisons, most baseline variables did not differ significantly across the study groups. However, for the comparison between the PCE group and the group with CD138-positive cell counts ≤4/HPF, assessment based on absolute standardized mean differences indicated meaningful imbalance in several covariates before weighting, particularly BMI, infertility duration, and endometrial thickness on transfer day; these imbalances were substantially reduced after IPTW (**Supplementary Figure 1**; **Supplementary Table 3**).

### 4.2 Prevalence of CE and antibiotic treatment regimens

Of the 2,885 women enrolled in the study, 1,609 (55.8%) had a CD138-positive plasma cell count of 0/HPF at initial evaluation, 790 (27.4%) had 1–4 CD138-positive plasma cells/HPF, and 486 (16.8%) had ≥5 CD138-positive plasma cells/HPF.

Among women with 1-4 CD138-positive plasma cells/HPF, 308 (39.0%) received oral antibiotics. All 486 women with CD138-positive plasma cells ≥5/HPF were prescribed oral antibiotics, and 364 (74.9%) were cured with CD138-positive/HPF ≤4 on follow-up endometrial biopsy, whereas 122 (25.1%) exhibited CD138-positive plasma cells ≥ 5/HPF.

After completion of a second course of oral antibiotic treatment, 69 of the 486 women (14.2%) were cured with CD138-positive/HPF ≤4, whereas 53 (10.9%) exhibited CD138-positive plasma cells ≥5/HPF. This second empirical regimen was administered to women with persistent CD138-positive plasma cells ≥5/HPF on repeat biopsy after initial treatment and should be distinguished from Group C in **Table 3**, which refers to women with CD138-positive/HPF =1-4 who did not receive antibiotic treatment. Overall, histological remission was achieved in 433 of 486 women (89.1%) after either the first or second antibiotic course.

### 4.3 Pregnancy outcomes among women with CD138-positive/HPF ≥5 who were cured after antibiotic treatment

Pregnancy outcomes were compared between women with CD138-positive plasma cell counts ≤4/HPF and those with ≥5/HPF who achieved remission after antibiotic treatment (CCE group). Pregnancy outcomes were comparable between the two groups, with no statistically significant differences in CPR (56.6% vs. 54.7%, P = 0.56), LBR (41.8% vs. 43.6%, P = 0.84), or EMR (10.3% vs. 8.9%, P = 0.45) (**Table 1**).

**Table 1:** Clinical characteristics and pregnancy outcomes of infertile women with CD138-positive/HPF ≤4 and those in the CCE group.

Characteristic and Outcome	CD138-positive/HPF ≤4	CCE	P Value
	(n =2399)	(n =433)	
Maternal age [years, M (Q1, Q3)]	33.8 (32.2, 35.1)	34.1 (31.5, 35.2)	0.52
Paternal age [years, M (Q1, Q3)]	34.5 (31.2, 36.3)	35.3 (32.1, 39.1)	0.41
Maternal BMI [kg/m2, M (Q1, Q3)]	22.1 (20.6, 23.2)	23.6 (21.0, 24.9)	0.28
Infertility duration [years, M (Q1, Q3)]	3.9 (1.9, 4.9)	4.7 (2.4, 6.6)	0.35
Aetiology, n (%)			
Tubal factor	1106 (46.1%)	206 (47.6%)	0.45
Ovulatory factor	383 (16.0%)	77 (17.8%)	
Male factor	606 (25.3%)	94 (21.6%)	
Others	304 (12.6%)	56 (13.0%)	
Basal FSH [IU/L, M (Q1, Q3)]	5.59 (3.92, 7.28)	6.01 (4.15, 8.25)	0.26
AMH [ng/mL, M (Q1, Q3)]	2.21 (0.95, 2.53)	2.62 (1.33, 2.83)	0.47
AFC (n)	8.45 (4.95, 8.95)	8.62 (3.95, 11.60)	0.32
Endometrium preparation protocols (%)			
HRT	849 (35.4%)	150 (34.6%)	0.53
Natural cycle	1301 (54.2%)	251 (58.0%)	
GnRH-a-HRT	249 (10.4%)	32 (7.4%)	
No. of embryos transferred	1.5 (1.0, 2.0)	1.5 (1.0, 2.0)	0.91
EMT on FET day [mm, M (Q1, Q3)]	8.5 (7.9, 9.5)	8.7 (7.9, 9.9)	0.63
Proportion of high-quality blastocysts transferred (%)	1382 (57.6%)	254 (58.7%)	0.87
Live birth rate	1003/2399 (41.8%)	189/433 (43.6%)	0.84
Clinical pregnancy rate	1358/2399 (56.6%)	237/433 (54.7%)	0.56
Early miscarriage rate	140/1358 (10.3%)	21/237 (8.9%)	0.45

BMI, body mass index; CCE, cured chronic endometritis; FSH, follicle stimulating hormone; AMH, Anti-Müllerian hormone; AFC, Antral follicle count; HRT, Hormone replacement treatment; GnRH-a-HRT, Gonadotropin-releasing hormone agonist combined with hormone replacement therapy; EMT, endometrial thickness; FET, Frozen embryo transfer.

### 4.4 Reproductive outcomes among women with PCE

Reproductive outcomes in the subsequent FET cycle were further compared between women with CD138-positive plasma cell counts ≤4/HPF and women with PCE. Women with CD138-positive plasma cell counts ≤4/HPF had significantly higher CPR (56.6% vs. 39.6%, P = 0.016) and LBR (41.8% vs. 28.3%, P = 0.034) than women with PCE. EMR was numerically higher in the PCE group than in the CD138-positive plasma cells ≤4/HPF group (19.0% vs. 10.3%), although the difference was not statistically significant (P = 0.328) (**Table 2**).

**Table 2:** Clinical characteristics and pregnancy outcomes of infertile women with CD138-positive/HPF ≤4 and those with PCE.

Characteristic and Outcome	CD138-positive/HPF ≤4	CCE	P Value
	(n =2399)	(n =53)	
Maternal age [years, M (Q1, Q3)]	33.8 (32.2, 35.1)	34.3 (31.0, 36.0)	0.53
Paternal age [years, M (Q1, Q3)]	34.5 (31.2, 36.3)	35.5 (31.9, 39.0)	0.41
Maternal BMI [kg/m2, M (Q1, Q3)]	22.1 (20.6, 23.2)	24.3 (21.5, 26.2)	0.33
Infertility duration [years, M (Q1, Q3)]	3.9 (1.9, 4.9)	4.9 (1.7, 6.6)	0.39
Aetiology, n (%)			
Tubal factor	1106 (46.1%)	24 (45.3%)	0.50
Ovulatory factor	383 (16.0%)	9 (17.0%)	
Male factor	606 (25.3%)	12 (22.6%)	
Others	304 (12.6%)	8 (15.1%)	
Basal FSH [IU/L, M (Q1, Q3)]	5.59 (3.92, 7.28)	5.51 (3.65, 7.75)	0.39
AMH [ng/mL, M (Q1, Q3)]	2.21 (0.95, 2.53)	2.24 (0.98, 2.56)	0.51
AFC (n)	8.45 (4.95, 8.95)	7.62 (3.45, 9.60)	0.67
Endometrium preparation protocols (%)			
HRT	849 (35.4%)	19 (35.8%)	0.72
Natural cycle	1301 (54.2%)	28 (52.9%)	
GnRH-a-HRT	249 (10.4%)	6 (11.3%)	
No. of embryos transferred	1.5 (1.0, 2.0)	1.6 (1.0, 2.0)	0.87
EMT on FET day [mm, M (Q1, Q3)]	8.5 (7.9, 9.5)	8.7 (8.0, 9.8)	0.48
Proportion of high-quality blastocysts transferred (%)	1382 (57.6%)	32 (60.4%)	0.73
Live birth rate	1003/2399 (41.8%)	15/53 (28.3%)	0.034
Clinical pregnancy rate	1358/2399 (56.6%)	21/53 (39.6%)	0.016
Early miscarriage rate	140/1358 (10.3%)	4/21 (19.0%)	0.328

BMI, body mass index; PCE, persistent chronic endometritis; FSH, follicle stimulating hormone; AMH, Anti-Müllerian hormone; AFC, Antral follicle count; HRT, Hormone replacement treatment; GnRH-a-HRT, Gonadotropin-releasing hormone agonist combined with hormone replacement therapy; EMT, endometrial thickness; FET, Frozen embryo transfer.

### 4.5 Pregnancy outcomes among women with CD138-positive/HPF =1-4

Participants were categorized into three cohorts based on endometrial CD138-positive plasma cell count and antibiotic exposure: Group A (0/HPF), Group B (1-4/HPF following antibiotic treatment), and Group C (1-4/HPF in the absence of antibiotic treatment). No significant differences were detected in LBR, CPR, or EMR among the groups (**Table 3**).

**Table 3:** General characteristics of women in Group A, Group B and Group C.

Variables	Group A (n = 1609)	Group B (n = 308)	Group C (n = 482)	P value
Maternal age [years, M (Q1 ,Q3)]	33.6 (32.0, 35.0)	34.3 (32.0, 35.0)	33.1 (29.0, 34.0)	0.48
Paternal age [years, M (Q1 ,Q3)]	34.3 (31.0, 36.0)	35.5 (32.0,39.3)	35.3 (32.0, 38.5)	0.37
Maternal BMI [kg/m2, M (Q1 ,Q3)]	22.2 (20.7, 23.3)	23.5 (21.1, 24.6)	23.8 (21.0, 24.9)	0.26
Infertility duration [years, M (Q1 ,Q3)]	4.0 (2.0, 5.0)	5.6 (2.5, 7.50)	3.9 (1.0, 5.5)	0.36
Aetiology, n (%)				
Tubal factor	686 (42.6%)	167 (54.2%)	253 (52.5%)	0.12
Ovulatory factor	279 (17.4%)	34 (11.0%)	70 (14.5%)	
Male factor	430 (26.7%)	75 (24.4%)	101 (21.0%)	
Others	214 (13.3%)	32 (10.4%)	58 (12.0%)	
Basal FSH [IU/L, M (Q1 ,Q3 )]	5.32 (3.91, 7.17)	6.06 (4.07, 7.27)	5.14 (3.88, 7.31)	0.14
AMH [ng/mL, M (Q1, Q3)]	2.06 (0.76,3.05)	1.77 (0.58,2.25)	2.65 (1.34,2.84)	0.39
AFC (n)	6.43 (2.00,10.00)	6.42 (3.00,7.00)	8.59 (4.00,11.50)	0.20
Endometrium preparation protocols (%)				
HRT	587 (36.5%)	103 (33.4%)	159 (33.0%)	0.101
Natural cycle	874 (54.3%)	158 (51.3%)	269 (55.8%)	
GnRH-a-HRT	148 (9.2%)	47 (15.3%)	54 (11.2%)	
No.of embryos transferred	1.5 (1.0, 2.0)	1.5 (1.0, 2.0)	1.6 (1.0, 2.0)	0.93
EMT on FET day [mm, M (Q1, Q3 )]	9.0 (8.2, 10.3)	8.6 (8.0, 9.6)	8.8 (8.0, 10.0)	0.38
Proportion of high-quality blastocysts transferred (%)	951 (59.1%)	181 (58.7%)	295 (61.2%)	0.67
Live birth rate	669/1609 (41.6%)	131/308 (42.5%)	203/482 (42.1%)	0.481
Clinical pregnancy rate	922/1609 (57.3%)	169/308 (54.9%)	267/482 (55.4%)	0.898
Early miscarriage rate	104/922 (11.3%)	14/169 (8.3%)	22/267 (8.2%)	0.345

BMI, body mass index; FSH, follicle stimulating hormone; AMH, Anti-Müllerian hormone; AFC, Antral follicle count; HRT, Hormone replacement treatment; GnRH-a-HRT, Gonadotropin-releasing hormone agonist combined with hormone replacement therapy; EMT, endometrial thickness; FET, Frozen embryo transfer; Group A: women with CD138+/HPF = 0; Group B: women with CD138+/HPF = 1-4 with antibiotic treatment; Group C: women with CD138+/HPF = 1-4 without antibiotic treatment.

### 4.6 Multivariable and IPTW-weighted analyses of pregnancy outcomes

After adjustment for maternal age, BMI, infertility duration, AMH, endometrial preparation protocol, number of embryos transferred, endometrial thickness on transfer day, and the proportion of high-quality blastocysts transferred, PCE remained significantly associated with poorer reproductive outcomes. Women with PCE had lower LBR (adjusted OR 0.63, 95% CI 0.48-0.82, P = 0.001) and CPR (adjusted OR 0.61, 95% CI 0.47–0.79, P < 0.001) than women with CD138-positive plasma cell counts ≤4/HPF (**Supplementary Tables 1, S2**). To further address the marked group-size imbalance between the PCE group and the CD138-positive/HPF ≤ 4 group, we performed a stabilized IPTW analysis using the same prespecified covariates. After weighting, baseline covariate balance was substantially improved, and all absolute SMDs were < 0.10 (**Supplementary Figure 1**; **Supplementary Table 3**). In the IPTW-weighted cohort, PCE remained significantly associated with lower CPR (weighted OR 0.65, 95% CI 0.47-0.89, P = 0.008), whereas the association with lower LBR remained directionally consistent but was attenuated to borderline statistical significance (weighted OR 0.73, 95% CI 0.53-1.02, P = 0.056) (**Supplementary Table 4**).

In contrast, no significant difference in LBR or CPR was observed between the CCE group and those with CD138-positive/HPF ≤4 (LBR: adjusted OR 1.02, 95% CI 0.86-1.21; CPR: adjusted OR 0.96, 95% CI 0.82-1.13) (**Supplementary Tables 1, S2**).

Similarly, among women with CD138-positive/HPF ≤4, antibiotic treatment was not associated with improved LBR (adjusted OR 0.95, 95% CI 0.76-1.19) or CPR (adjusted OR 1.03, 95% CI 0.83-1.28) after multivariable analysis (**Supplementary Tables 1, S2**).

## 5 Discussion

In this large retrospective cohort of women undergoing subsequent FET after FIF, we found that women with ≥5 CD138-positive plasma cells/HPF who achieved histological remission after treatment had pregnancy outcomes comparable to those of women with ≤4 CD138-positive plasma cells/HPF. In contrast, among women with CD138-positive plasma cells of 1–4/HPF, antibiotic exposure was not associated with improved reproductive outcomes.

A major contribution of this study is its clarification of the diagnostic threshold for CE, a subject of ongoing debate. Histopathological identification of plasma cell infiltration within the endometrial stroma is currently regarded as the gold standard for diagnosing CE (6). However, no universally accepted quantitative threshold has been established to standardize this diagnosis (7). Several research groups have proposed expanded histological criteria for CE, defining it as a CD138-positive cell count of ≥1/HPF (19–21). Moreover, a histological grading system has been introduced, classifying 1-4 CD138-positive/HPF as mild CE (15, 21). Although antibiotic therapy has often been reported to improve reproductive outcomes in selected CE populations, our findings showed no significant benefit of antibiotic treatment among women with CD138-positive/HPF = 1-4. This interpretation is consistent with recent evidence suggesting that antibiotic treatment may not improve pregnancy outcomes in patients with mild CE undergoing IVF (22). These findings corroborate previous reports indicating that a low CD138-positive plasma cell count (e.g., 1-4/HPF) exhibits limited clinical relevance (15, 21, 23). However, existing evidence is limited by small sample sizes and a predominant focus on patients with RIF. Our study corroborates and extends these observations. Given the ongoing controversy regarding the diagnostic threshold for CE, this convergence strengthens the argument against reflexive antibiotic prescribing based solely on minimal CD138-positive plasma cell infiltration. Consequently, defining CE solely on the basis of ≥1 CD138-positive plasma cell/HPF is likely to result in overdiagnosis.

In the present study, CE, defined as CD138-positive plasma cells ≥5/HPF, was identified in 16.8% of women evaluated after FIF. Importantly, all women who met this prespecified histological criterion received standardized empirical antibiotic treatment as part of routine clinical management. This treatment approach reflected a strict ethical consideration in clinical practice: once CE had been histologically confirmed in women preparing for a subsequent embryo transfer, deliberately withholding treatment solely to create an untreated comparator group was considered ethically inappropriate, given the potential adverse reproductive implications of persistent endometrial inflammation. Consequently, a treated-versus-untreated comparison among women with CE could not be performed in the present retrospective cohort. Nevertheless, the structured post-treatment follow-up pathway enabled us to evaluate clinically relevant post-treatment CE status before subsequent FET. Overall, 433 of 486 women with initial CE were classified as having CCE after one or two antibiotic courses, whereas 53 were classified as having PCE. Women with CCE had subsequent FET outcomes comparable with those of women with CD138-positive plasma cells ≤4/HPF at initial evaluation, whereas women with PCE had poorer reproductive outcomes. Therefore, the present findings do not establish that antibiotic treatment itself causally improves reproductive outcomes; rather, they indicate that post-treatment CE reassessment may provide clinically meaningful prognostic information for women undergoing subsequent FET after FIF.

We also acknowledge that CE is etiologically heterogeneous and that plasma cell burden does not, by itself, define the underlying pathogen profile. In principle, pathogen identification and susceptibility-guided treatment may help individualize management when feasible. However, such microbiological characterization, including viral evaluation, was not systematically available in the present retrospective cohort. Therefore, our findings should be interpreted as reflecting outcomes under a standardized empirical treatment pathway, rather than evidence that CD138 expression alone is sufficient to determine the optimal anti-infective regimen. Previous studies focusing on RIF, RPL, or unexplained infertility have also reported that CE, particularly when defined using higher CD138-positive plasma cell thresholds, is associated with impaired reproductive outcomes. Some studies further suggest that women who achieve CE cure after antibiotic treatment may have reproductive outcomes approaching those of women without CE, although findings vary across populations and study designs (11, 15, 20, 23, 24). To our knowledge, this study is among the largest cohorts specifically evaluating CE and post-treatment CE status in women experiencing FIF, before they meet conventional criteria for RIF. Research focused on this population has important clinical implications: the timely identification of underlying etiologies-including CE-and the implementation of targeted interventions could improve pregnancy outcomes. These findings may support a shift in strategy in the management of implantation failure from reactive treatment after repeated failure to proactive evaluation and intervention at an earlier stage.

In the present study, PCE was associated with impaired pregnancy outcomes in subsequent FET cycles. This adverse association was observed in the crude comparison, remained evident after multivariable adjustment, and was further supported by the IPTW-weighted sensitivity analysis. After weighting, the association with lower CPR remained statistically significant, whereas the association with lower LBR remained directionally consistent but was attenuated to borderline significance, suggesting that the observed findings are unlikely to be explained solely by measured baseline imbalance. This finding is corroborated by multiple studies (10, 25, 26), including recent evidence showing that the presence of CE may adversely influence pregnancy outcomes in infertile women with mild endometriosis (27). Our findings extend this conceptual framework to the clinically distinct scenario of FIF, suggesting that PCE may define a clinically meaningful risk state even before patients meet conventional criteria for recurrent implantation failure. From a mechanistic perspective, CE may alter the endometrial microenvironment through dysregulation of inflammatory mediators and compromised decidualization, thereby impairing implantation and early placentation.

From a biological standpoint, the observed association between PCE and adverse reproductive outcomes aligns with current evidence regarding the pathophysiological dynamics of the endometrial microenvironment. We also acknowledge that CE is etiologically heterogeneous, and different underlying causes may contribute differently to implantation failure and subsequent reproductive outcomes. However, because etiologic characterization was not systematically available in this retrospective cohort, the present study was not able to stratify outcomes according to specific CE etiologies. CE is predominantly attributable to dysbiotic intrauterine microbial colonization, as shown by microbiome studies (28, 29) and further corroborated by high histological cure rates following antibiotic therapy (30). It triggers a distinct pro-inflammatory endometrial response, elevating IL-11, CXCL4, TNF-α, and IL-6-which disrupts immune tolerance and impairs trophoblast function, thereby reducing receptivity (6, 21, 31). Furthermore, CE impairs decidualization and blunts progesterone-driven gene expression in stromal cells, directly compromising embryo implantation and early placentation (32, 33). In PCE, persistent plasma cells despite treatment may suggest a microbiome-immune “lock-in” state, potentially due to antibiotic resistance or dysbiosis-associated chronic inflammation-explaining the markedly reduced pregnancy success in this subgroup. Nevertheless, because specific bacterial, chlamydial, mycoplasmal, or viral etiologies were not systematically assessed, these mechanistic interpretations remain indirect and should be viewed with caution. Therapeutic interventions informed by either pathogen-specific antimicrobial susceptibility profiles or comprehensive assessment of the endometrial immune microenvironment hold promise for enhancing pregnancy outcomes in patients with PCE. However, the present study was not designed to evaluate pathogen-directed treatment strategies, because microbiological and virological characterization were not systematically available in this retrospective cohort. These findings suggest that CD138-positive plasma cell counts ≥5/HPF may identify a clinically relevant inflammatory state in this population, whereas counts ≤4/HPF were not associated with impaired reproductive outcomes in the present cohort.

Our findings underscore the critical importance of antibiotic stewardship within the field of reproductive medicine. From a safety and public health perspective, unnecessary antibiotic exposure can contribute to antimicrobial resistance and may disrupt genital tract microbial communities, both of which could plausibly influence reproductive outcomes and broader health risks (15, 28). Our findings should not be interpreted as supporting indiscriminate antibiotic use in all women after FIF. Rather, within the ethically mandated clinical pathway-where women meeting the prespecified histological criteria for CE received treatment-post-treatment reassessment effectively distinguished those who achieved a cure (CCE) from those with persistent disease (PCE), with the latter representing a subgroup at risk for poorer subsequent FET outcomes. Future prospective studies should focus on optimizing diagnostic thresholds, treatment regimens, and follow-up strategies without compromising ethically appropriate care for women with confirmed CE. Moreover, intrauterine antibiotic infusion is being actively investigated as an alternative or adjunctive strategy for PCE, though current evidence remains limited and inconsistent (34, 35).

This study has several notable strengths. First, this large-scale cohort study evaluated CE in women with FIF, enabling assessment of the rationale for early CE screening and of reproductive outcomes according to post-treatment CE status under a standardized empirical management pathway, unlike most existing studies, which focus primarily on RIF or RPL. Second, all endometrial samples were obtained during the follicular phase (within one week after the end of menstruation), thereby minimizing inter-cycle variability in CE prevalence due to sampling timing (36, 37). Finally, we restricted analysis to FET cycles, thereby eliminating confounding by controlled ovarian stimulation (COH) protocols, which directly alter endometrial receptivity in fresh cycles (38).

However, several limitations merit consideration. First, given its retrospective, single-center design, the possibility of residual confounding cannot be entirely eliminated despite the rigorous application of multivariable adjustment and IPTW. Second, although IPTW significantly improved the balance of measured baseline characteristics between the PCE and initial CD138-positive (≤4/HPF) groups, the PCE subgroup remained relatively small. This limited sample size may have constrained the statistical precision of our weighted effect estimates and the overall stability of the propensity score model. Third, confounding by indication remains an inherent consideration within the low-positive CD138 subgroup (1–4/HPF), where antibiotic administration was not protocol-mandated but instead individualized based on clinician discretion and shared decision-making. Fourth, because all patients meeting our diagnostic threshold for CE (CD138 ≥5/HPF) received standardized antibiotic treatment as part of routine clinical care, an untreated CE comparator group was unavailable. This lack of an untreated control group reflects a necessary ethical imperative in routine patient care rather than an omission in study design or follow-up. Consequently, our findings cannot definitively establish a direct causal relationship between the antibiotic therapy itself and the subsequent reproductive variations; rather, the primary value of these data lies in demonstrating the prognostic utility of post-treatment histological reassessment within an ethically sound, standardized clinical pathway. Fifth, because the initial diagnostic biopsy was obtained under direct hysteroscopic visualization using forceps, whereas the post-treatment histological reassessment relied on Pipelle suction curettage, we cannot completely exclude sampling-related variability in plasma cell quantification or potential procedure-induced effects from serial endometrial manipulation. In this regard, randomized evidence in women with RIF has suggested that mechanical endometrial scratching via a Pipelle catheter does not significantly improve implantation or clinical pregnancy rates compared with a sham procedure (39). While that trial evaluated a distinct clinical intent and patient population, it offers highly relevant context for interpreting the potential independent impact of endometrial instrumentation. Because every patient in our cohort underwent a baseline biopsy and those undergoing post-treatment reassessment were instrumented a second time, this retrospective design precludes us from isolating the independent contribution of mechanical manipulation to subsequent reproductive outcomes. Finally, comprehensive endometrial microbial profiling and virological characterization were not systematically available in this cohort; therefore, we could not evaluate whether distinct etiological phenotypes are differentially associated with initial implantation failure or subsequent pregnancy outcomes.

Prospective, well-controlled studies are required to validate these findings and to further refine diagnostic thresholds, treatment strategies, and post-treatment assessment pathways for CE following FIF. Furthermore, future investigations incorporating standardized microbiological and, where applicable, virological profiling may help determine whether specific etiologies are associated with persistent endometrial inflammation or adverse reproductive outcomes. Crucially, longitudinal follow-up of women from the initial FIF evaluation through subsequent embryo transfer cycles will be essential. Such studies will clarify whether CE identified early in the clinical trajectory-particularly PCE refractory to initial treatment-drives recurrent implantation failure and subsequent progression to RIF. Ultimately, these lines of evidence will support more individualized, stage-specific management strategies for women navigating implantation failures.

## 6 Conclusion

Among women with FIF, CD138-positive plasma cell counts ≤4/HPF were not associated with impaired pregnancy outcomes. Furthermore, after standard antibiotic therapy, women with ≥5 CD138-positive plasma cells/HPF had pregnancy outcomes comparable to those of women with ≤4 CD138-positive plasma cells/HPF. In contrast, PCE was associated with poorer reproductive outcomes.

## Data Availability

The original contributions presented in the study are included in the article/**Supplementary Material**. Further inquiries can be directed to the corresponding author.

## Supplementary material

Supplementary Figure 1 Covariate balance before and after inverse probability of treatment weighting (IPTW). A Love plot showing the absolute standardized mean differences (SMDs) of prespecified baseline covariates before and after stabilized IPTW for the comparison between women with persistent chronic endometritis (PCE) and those with CD138-positive/HPF ≤4. The vertical dashed line indicates the threshold for acceptable covariate balance, defined as an absolute SMD <0.10. After IPTW, all covariates achieved acceptable balance. The three levels of endometrial preparation protocol, including HRT, NC, and GnRH-a-HRT, are displayed separately. IPTW, inverse probability of treatment weighting; SMD, standardized mean difference; PCE, persistent chronic endometritis; HPF, high-power field; BMI, body mass index; AMH, anti-Müllerian hormone; HRT, hormone replacement therapy; NC, natural cycle; GnRH-a-HRT, gonadotropin-releasing hormone agonist combined with hormone replacement therapy.
