# Supplementary figures and images for: Early screening and post-treatment chronic endometritis in subsequent frozen embryo transfer cycles among women with first implantation failure: a retrospective cohort study
Source: Front Endocrinol (Lausanne). 2026 Jul 8;17:1811073. doi: 10.3389/fendo.2026.1811073 (PMC13388128; doi:10.3389/fendo.2026.1811073)

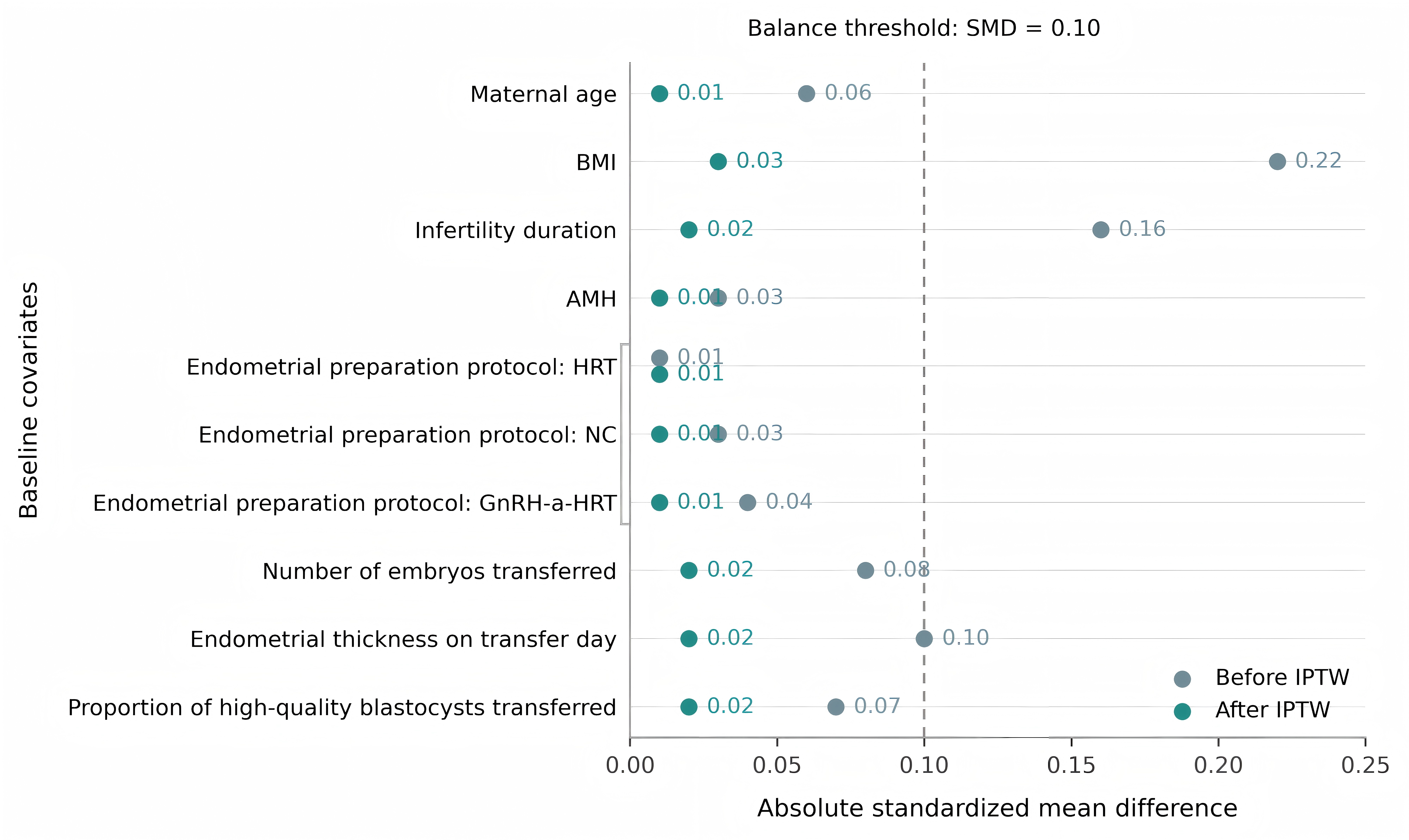

Supplement: Supplementary Figure 1 — Covariate balance before and after inverse probability of treatment weighting (IPTW). A Love plot showing the absolute standardized mean differences (SMDs) of prespecified baseline covariates before and after stabilized IPTW for the comparison between women with persistent chronic endometritis (PCE) and those with CD138-positive/HPF ≤4. The vertical dashed line indicates the threshold for acceptable covariate balance, defined as an absolute SMD <0.10. After IPTW, all covariates achieved acceptable balance. The three levels of endometrial preparation protocol, including HRT, NC, and GnRH-a-HRT, are displayed separately. IPTW, inverse probability of treatment weighting; SMD, standardized mean difference; PCE, persistent chronic endometritis; HPF, high-power field; BMI, body mass index; AMH, anti-Müllerian hormone; HRT, hormone replacement therapy; NC, natural cycle; GnRH-a-HRT, gonadotropin-releasing hormone agonist combined with hormone replacement therapy. [file Image1.jpeg]
