# Supplementary material for: Early screening and post-treatment chronic endometritis in subsequent frozen embryo transfer cycles among women with first implantation failure: a retrospective cohort study
Source: Front Endocrinol (Lausanne). 2026 Jul 8;17:1811073. doi: 10.3389/fendo.2026.1811073 (PMC13388128; doi:10.3389/fendo.2026.1811073)
Supplement: Supplementary file 2 [file Table1.doc]

Supplementary Table 1. Multivariable logistic regression of live birth rate following FET

| Exposure | Adjusted OR (95% CI) | P value |
| --- | --- | --- |
| Maternal age | 0.96 (0.94-0.98) | <0.001 |
| BMI | 0.99 (0.97-1.02) | 0.56 |
| infertility duration | 0.97 (0.95-0.99) | 0.012 |
| AMH | 1.07 (1.03-1.12) | 0.001 |
| Endometrial preparation protocol |  |  |
| NC | Ref. |  |
| HRT | 0.93 (0.80-1.08) | 0.33 |
| GnRH-a-HRT | 0.96 (0.81-1.13) | 0.62 |
| Number of embryos transferred | 1.38 (1.16-1.64) | <0.001 |
| Endometrial thickness on transfer day | 1.11 (1.06-1.16) | <0.001 |
| proportion of high-quality blastocysts transferred | 1.05 (1.02-1.08) | 0.002 |
| PCE vs CD138 ≤ 4 (ref) | 0.63 (0.48-0.82) | 0.001 |
| CCE vs CD138 ≤ 4 (ref) | 1.02 (0.86-1.21) | 0.81 |
| Antibiotic in CD138 1-4 (with vs without antibiotic treatment) (ref) | 0.95 (0.76-1.19) | 0.64 |

Notes: Adjusted models included: maternal age, BMI, infertility duration, AMH, endometrial preparation protocol, number of embryos transferred, endometrial thickness on transfer day, and proportion of high-quality blastocysts transferred.

Abbreviations: NC, natural cycle; HRT, hormone replacement therapy; GnRH-a-HRT, GnRH-a combined with hormone replacement therapy; BMI, Body mass index; AMH, anti-Müllerian hormone; OR, odds ratio; CI, confidence interval.
