# Supplementary material for: Early screening and post-treatment chronic endometritis in subsequent frozen embryo transfer cycles among women with first implantation failure: a retrospective cohort study
Source: Front Endocrinol (Lausanne). 2026 Jul 8;17:1811073. doi: 10.3389/fendo.2026.1811073 (PMC13388128; doi:10.3389/fendo.2026.1811073)
Supplement: Supplementary file 3 [file Table2.doc]

Supplementary Table 2. Multivariable logistic regression of clinical pregnancy rate following FET

| Exposure | Adjusted OR (95% CI) | P value |
| --- | --- | --- |
| Maternal age | 0.95 (0.93-0.97) | <0.001 |
| BMI | 0.99 (0.97-1.01) | 0.38 |
| infertility duration | 0.98 (0.96-1.00) | 0.041 |
| AMH | 1.06 (1.02-1.10) | 0.002 |
| Endometrial preparation protocol |  |  |
| NC | Ref. |  |
| HRT | 0.95 (0.83-1.09) | 0.47 |
| GnRH-a-HRT | 0.98 (0.84-1.15) | 0.81 |
| Number of embryos transferred | 1.31 (1.12-1.53) | 0.001 |
| Endometrial thickness on transfer day | 1.10 (1.06-1.14) | <0.001 |
| proportion of high-quality blastocysts transferred | 1.06 (1.03-1.09) | <0.001 |
| PCE vs CD138 ≤ 4 (ref) | 0.61 (0.47-0.79) | <0.001 |
| CCE vs CD138 ≤ 4 (ref) | 0.96 (0.82-1.13) | 0.62 |
| Antibiotic in CD138 1-4 (with vs without antibiotic treatment) (ref) | 1.03 (0.83-1.28) | 0.78 |

Notes: Adjusted models included: maternal age, BMI, infertility duration, AMH, endometrial preparation protocol, number of embryos transferred, endometrial thickness on transfer day, and proportion of high-quality blastocysts transferred.

Abbreviations: NC, natural cycle; HRT, hormone replacement therapy; GnRH-a-HRT, GnRH-a combined with hormone replacement therapy; BMI, Body mass index; AMH, anti-Müllerian hormone; OR, odds ratio; CI, confidence interval.
