# Supplementary material for: Early screening and post-treatment chronic endometritis in subsequent frozen embryo transfer cycles among women with first implantation failure: a retrospective cohort study
Source: Front Endocrinol (Lausanne). 2026 Jul 8;17:1811073. doi: 10.3389/fendo.2026.1811073 (PMC13388128; doi:10.3389/fendo.2026.1811073)
Supplement: Supplementary file 4 [file Table3.doc]

Supplementary Table S3. Baseline covariate balance before and after inverse probability of treatment weighting (IPTW) in women with persistent chronic endometritis (PCE) and those with CD138-positive/HPF ≤ 4

| Covariate | PCE (unweighted) | CD138-positive/HPF ≤4 (unweighted) | Absolute SMD before IPTW | PCE (weighted) | CD138-positive/HPF ≤4 (weighted) | Absolute SMD after IPTW |
| --- | --- | --- | --- | --- | --- | --- |
| Maternal age, years | 34.3 (31.0, 36.0) | 33.8 (32.2, 35.1) | 0.06 | 34.1 (31.8, 35.7) | 33.9 (32.1, 35.5) | 0.01 |
| BMI, kg/m² | 24.3 (21.5, 26.2) | 22.1 (20.6, 23.2) | 0.22 | 22.7 (21.0, 24.5) | 22.6 (20.8, 24.3) | 0.03 |
| Infertility duration, years | 4.9 (1.7, 6.6) | 3.9 (1.9, 4.9) | 0.16 | 4.1 (1.8, 5.7) | 4.0 (1.9, 5.5) | 0.02 |
| AMH, ng/mL | 2.24 (0.98, 2.56) | 2.21 (0.95, 2.53) | 0.03 | 2.22 (0.96, 2.54) | 2.19 (0.95, 2.53) | 0.01 |
| Endometrial preparation protocol: HRT, % | 35.8 | 35.4 | 0.01 | 35.4 | 35.1 | 0.01 |
| Endometrial preparation protocol: NC, % | 52.9 | 54.2 | 0.03 | 53.2 | 53.4 | 0.01 |
| Endometrial preparation protocol: GnRH-a-HRT, % | 11.3 | 10.4 | 0.04 | 11.1 | 11.2 | 0.01 |
| Number of embryos transferred | 1.6 (1.0, 2.0) | 1.5 (1.0, 2.0) | 0.08 | 1.56 (1.0, 2.0) | 1.55 (1.0, 2.0) | 0.02 |
| Endometrial thickness on transfer day, mm | 8.7 (8.0, 9.8) | 8.5 (7.9, 9.5) | 0.10 | 8.62 (7.9, 9.6) | 8.59 (7.9, 9.5) | 0.02 |
| Proportion of high-quality blastocysts transferred, % | 60.4 | 57.6 | 0.07 | 58.7 | 58.4 | 0.02 |

Notes: Propensity scores were estimated using a logistic regression model including maternal age, BMI, infertility duration, AMH, endometrial preparation protocol, number of embryos transferred, endometrial thickness on transfer day, and the proportion of high-quality blastocysts transferred. Stabilized inverse probability of treatment weighting (IPTW) was applied. Covariate balance was assessed using absolute standardized mean differences (SMDs), with an absolute SMD <0.10 considered indicative of acceptable balance. Continuous variables are presented as median (Q1, Q3) for descriptive purposes, and categorical variables are presented as percentages. Covariate balance was evaluated primarily on the basis of absolute SMDs.

Abbreviations: PCE, persistent chronic endometritis; HPF, high-power field; BMI, body mass index; AMH, anti-Müllerian hormone; HRT, hormone replacement therapy; NC, natural cycle; GnRH-a-HRT, gonadotropin-releasing hormone agonist combined with hormone replacement therapy; IPTW, inverse probability of treatment weighting; SMD, standardized mean difference.
