# Supplementary material for: Early screening and post-treatment chronic endometritis in subsequent frozen embryo transfer cycles among women with first implantation failure: a retrospective cohort study
Source: Front Endocrinol (Lausanne). 2026 Jul 8;17:1811073. doi: 10.3389/fendo.2026.1811073 (PMC13388128; doi:10.3389/fendo.2026.1811073)
Supplement: Supplementary file 5 [file Table4.doc]

Supplementary Table S4. IPTW-weighted associations between persistent chronic endometritis (PCE) and reproductive outcomes following frozen embryo transfer

| Outcome | Weighted OR (95% CI) | P value |
| --- | --- | --- |
| Live birth rate | 0.73 (0.53–1.02) | 0.056 |
| Clinical pregnancy rate | 0.65 (0.47–0.89) | 0.008 |

Notes: Weighted logistic regression with robust variance estimation was performed after stabilized inverse probability of treatment weighting (IPTW). Women with CD138-positive/HPF ≤4 served as the reference group. Propensity scores were estimated using maternal age, BMI, infertility duration, AMH, endometrial preparation protocol, number of embryos transferred, endometrial thickness on transfer day, and the proportion of high-quality blastocysts transferred.

Abbreviations: IPTW, inverse probability of treatment weighting; PCE, persistent chronic endometritis; OR, odds ratio; CI, confidence interval; HPF, high-power field.
